# Probabilistic Conversion of the Compressive Strength of Cubes to Cylinders of Natural and Recycled Aggregate Concrete Specimens

**DOI:** 10.3390/ma12020280

**Published:** 2019-01-16

**Authors:** João Nuno Pacheco, Jorge de Brito, Carlos Chastre, Luís Evangelista

**Affiliations:** 1 CERIS, Department of Civil Engineering, Architecture and Georresources, Instituto Superior Técnico, Universidade de Lisboa, Av. Rovisco Pais, 1049-001 Lisbon, Portugal; joaonpacheco@ist.utl.pt; 2 CERIS, DECivil, Faculdade de Ciências e Tecnologia, Universidade Nova de Lisboa, 2829-516 Caparica, Portugal; chastre@fct.unl.pt; 3 CERIS, ISEL, Department of Civil Engineering, Instituto Superior de Engenharia de Lisboa, Rua Conselheiro Emídio Navarro, 1, 1959-007 Lisbon, Portugal; evangelista@dec.isel.ipl.pt

**Keywords:** concrete testing, structural codes, probabilistic analysis, shape effects, recycled concrete

## Abstract

This paper investigates the effect of recycled coarse aggregate incorporation on the relationship between 150 mm cubic and Փ 150 mm cylindrical compressive strength (the reference strength of standards) by comparing data from recycled and natural aggregate concrete compositions in which both cubes and cylinders were tested. A conversion factor from cubic to cylindrical strength is proposed in two versions: A deterministic and a probabilistic one. Such factor has not been studied before and researchers have been converting cubic data as if natural aggregate concrete were tested. The probabilistic factor is intended for reliability analyses on the structural behaviour of recycled aggregate concrete using data from laboratory cube tests. It was found that the incorporation of recycled coarse aggregates sourced from concrete waste significantly decreases the expected value of the factor but the factor’s scatter is relatively unaffected.

## 1. Introduction

In the last decades, the viability of recycled aggregate concrete (RAC) structural elements has been extensively investigated, especially in what concerns the incorporation of recycled aggregates (RA) sourced from concrete waste. Most of these studies are laboratory experiments following standardized procedures, with few applications in actual concrete industry environments [1,2,3]. Standards define the conditions for the assessment of most material properties of concrete. Curing, compaction, specimen geometry, test equipment, and load application rates are specified, intending that specimens produced in different regions or by different agents are comparable. However, because of long-standing regional tradition and practicability, different regions test material properties in different specimen shapes and sizes.

In some countries (e.g., Portugal, United Kingdom) the compressive strength is commonly tested on 150 mm cubic specimens; in others (e.g., the United States of America, Australia) on cylinders (either Փ 100 mm × 200 mm or Փ 150 mm × 300 mm). Structural codes (ACI318-14 [4]; Eurocode 2 [5]), define the compressive strength and subsequent constitutive models in terms of Փ 150 mm × 300 mm cylinders. This poses different problems since the compressive strength depends on the size and shape of the specimen tested [6]. A proper comparison of test data from different researches is hindered, as well as when onsite assessments made by coring are compared with quality control tests on cubes.

Standards approach this problem with relationships that convert the compressive strength of different specimens’ dimensions and shapes [7,8,9]. These relationships are estimates with some scatter, since shape and size effects are related to concrete composition and no factor can cover all concrete compositions accurately.

Since the mesostructure of RAC differs from that of natural aggregate concrete (NAC) and RA differ in elastic and inelastic properties from natural aggregates (NA) [10,11], the conversion factors of NAC may not be applicable to RAC. Moreover, since RA are more heterogeneous than NA, conversion factors may also be less precise. This paper appraises all available data known to the authors on the compressive strength of RAC compositions in which 150 mm cubes and standard Փ 150 cylinders are simultaneously tested and evaluates whether the conversion from 150 mm cubes to standard Փ 150 cylinders assumed in Eurocode 2 [5] and EN-206 [8] is suitable for RAC.

## 2. Research Significance and Goals

This study appraises compressive strength test data on natural aggregate concrete (NAC) and analogue RAC specimens, intending to:

- Evaluate the suitability of the cubic to cylindrical NAC conversion factors of Eurocode 2 and EN-206 for RAC with recycled aggregates (RA) sourced from concrete waste;
- Propose a conservative deterministic factor for RAC cube to cylinder strength conversions;
- Propose a probability distribution suitable for RAC strength conversions allowing the use of data from RAC cubic specimens for reliability analyses, including RAC code calibrations.

Future research on RAC should concern its acknowledgment as a structural material [12]. This includes the definition of constitutive equations that relate the compressive strength with the other properties used in concrete codes and the evaluation of the accuracy of test data on RAC when compared with actual structural applications. Constitutive equations have been defined by meta-analyses [13,14], which often resort to conversion factors to group data on a given property taken from tests on specimens with different geometry. Most compressive strength tests on RAC have been performed in 150 mm cubes and a proper conversion factor from cubic to cylindrical strength is necessary. Since a conversion factor will be sensitive to scatter of the results, a probabilistic evaluation of its modelling errors is necessary for reliability analyses and RAC code calibration procedures.

## 3. Background

Concrete under uniaxial compression follows an approximately elastic stress-strain relationship until stresses of about 30%–40% of the peak stress. Until then, damage is concentrated on the initial micro cracks of the interfacial transition zone (ITZ) between coarse aggregates and the cementitious matrix. Afterwards, micro cracks start developing from the ITZ towards the cementitious matrix and concrete stiffness decreases [15]. Stress-strain is no longer approximately elastic. When stresses surpass about 75% of the peak stress, cracks start coalescing on a fracture process zone and stiffness greatly decreases. After the peak stress, crack development and coalescence are very significant and a failure surface forms in a brittle manner [16,17].

Micro crack development happens mostly in the fracture process zone, whose size is non-negligible compared to that of the concrete element [18]. This results in a size effect explained by fracture mechanics [18]: The energy necessary for micro crack progression is not as dependent on specimen size and shape as the elastic energy stored in a specimen under a given load. In a large specimen, the energy necessary for micro crack progression is reached for lower loads than on a smaller one since the elastic energy increases. As a result, the peak stress of larger specimens decreases [17,19].

Statistical size effects also contribute to geometry dependence: since the fracture process zone is a localized region that limits strength, the larger the specimen size, the most likely a weak region is to be found [18].

The natural consequence of shape and size effects is that the concrete’s compressive behaviour is a structural property, rather than a material one [20,21,22], hence specimen shape will affect the compressive strength of laboratory tests.

The friction between the specimen and the plates of the testing equipment caused by Poisson effects in the secondary loading directions puts the specimen under a multiaxial stress-state and is also a cause for shape effects. Specimens with lower slenderness have higher peak stresses, because they benefit the most from this confinement [23].

There is no consensus for NAC conversion factors [24]. Nevertheless, since codes rely on the compressive strength of Փ 150 cylinders for design, and testing in Europe is often made on 150 mm cubes, Eurocode 2 and EN206 define the strength class in terms of cubic and cylindrical strength (the underlying cube to cylinder strength factor (*K*) depends on the compressive strength and varies from 0.78 to 0.83). A constant *K* of 0.80 has been used widely to convert 150 mm cubic to Փ 150 cylindrical strength [25].

## 4. RAC Size Effects

Since shape and size effects are dependent on material heterogeneity and fracture properties, RAC conversion factors may differ from those used for NAC. RA are more heterogeneous than NA, have different interfacial transition zones (ITZ) [26], and different elastic and inelastic properties [11,27] when compared to NA. Several studies on the mechanical performance of RAC have been performed, but only a few are related to fracture mechanisms, crack development, and softening.

These aspects may change the pattern of damage during uniaxial compression described in the background section, since they are related to micro crack, damage development, and stress-paths:

- The higher heterogeneity of the RA may contribute to higher fracture energies in low to medium strength concrete (when compared to NAC with the same compressive strength), since aggregate heterogeneity is related to tortuous fracture surfaces [16];
- The lower stiffness of the RA results in higher strains at peak stress [28], changing stress-strain relationships, stress paths, and damage pattern;
- The lower stiffness of the RA also results in lower E of RAC, increasing the friction between the test plates and the specimen (the reason for the higher confinement of cubes in comparison to cylinders) [29];
- For the same compressive strength of NAC, the relative strength of the ITZ of RAC is higher, since the hydration products of the ITZ tend to be denser and “nailed” to the attached mortar of the RA [11,30], as long as the RA are not soaked prior to mixing—a two-stage mixing approach [31] is recommended to offset workability losses without compromising this beneficial effect.

Supporting the fourth claim, Carpenteri et al. [22] argued in a study on NAC that coarse aggregates affect the stress and strain fields greatly and that the characteristics of the ITZ between coarse aggregates and mortar is instrumental in the development of the cracking pattern. Guo et al. [27] and Li et al. [32] have reported that when coarse NA are replaced by coarse RA, the failure surface will change from mostly comprising ITZ failures towards trans-aggregate fractures. This change in post-cracking load paths will affect the fracture process zone and will probably affect the relationship between cylindrical and cubic strength. For high-strength concrete, trans-aggregate fracture surfaces are expected, irrespective of RA incorporation [33], and failure is brisker [16].

In the investigation of Guo et al. [27], the development of the failure process zone on RAC with coarse RA sourced from concrete was tested. The authors reported that RA incorporation resulted in premature and faster development of the failure process zone and that the number of trans-aggregate failures on the failure surface increased when RA were incorporated. The authors also reported that RA incorporation leads to diffused micro cracking along the concrete elements.

The only study known to the authors that specifically investigates the relationship between the compressive strength of RAC cubes and cylinders was performed by Kheder and Al-Windah [29]. The authors produced mixes with similar mix design and compressive strength of NAC and RAC with full incorporation of coarse RA sourced from concrete waste (CRAC). The authors found that CRA incorporation decreases *K*: the values of *K* for NAC were in the range of 0.81–0.92, whilst those of RAC varied between 0.71 and 0.84. Kheder and Al-Windah [29] found that *K* is best related to the Young’s modulus than to the compressive strength, but studying and/or defining *K* in terms of the Young’s modulus is not practical. Since code predictions of the Young’s modulus based on the compressive strength have a high scatter [34], reliable *K* assessments based on the Young’s modulus would require the specific testing of this property.

## 5. Appraisal and Statistical Analysis of Data

This study concerns the conversion of the compressive strength of 150 mm cubes to Փ 150 mm × 300 mm cylinders only. Despite the comprehensive state-of-the-art on the compressive strength of RAC, research on the compressive strength of RAC specimens with other sizes and shape lacks a sample size that would allow a proper statistical evaluation. Also, because of sample size, only RAC data concerning the full incorporation of coarse aggregates sourced from concrete waste (CRA) was collected.

Table 1 shows the investigations appraised for the two datasets analysed, one with NAC and another with CRAC data. All mixes concern concrete with similar (and common) maximum aggregate diameter and the number of samples tested is similar between studies.

All data on NAC came from studies where CRAC was also evaluated (hence CRAC and NAC mixes had similar mix design and materials), minimizing possible causes of bias. Including NAC mixes from investigations where CRAC was not tested would result in:

- Comparing datasets having concrete mixes with different mix design, materials, and execution conditions (in this study all NAC compositions are paired with analogue RAC compositions);
- Including NAC tests that would not be paired with RAC experiments performed with the same testing equipment and protocols, such factors such as different roughness of test plates (which influences specimen/plate friction), cylinder capping method, and mould material.

As seen in Table 2, the distribution of w/b ratios, binder contents, and cubic compressive strength of the NAC and CRAC mixes is similar. Binders were either ordinary Portland cement (OPC), or blends of OPC with either fly ash—two NAC and two CRAC mixes of [38], or ground granulated furnace slag—one NAC and one CRAC mix from [36]. High-range water admixtures were used in some of the compositions of [29,38]. Only 28-day test data were collected.

The 150 mm cube to Փ 150 mm cylinder conversion factor (*K*) of each mix was calculated using Equation (1). Most authors tested 3 to 5 specimens of each shape.

(1) $$K=f_{c,cyl\;\emptyset 150}/f_{c,cub\;150}$$

where:

- $f_{c,cyl\;\emptyset 150}$ is the compressive strength tested in Փ 150 mm × 300 mm cylinders;
- $f_{c,cub\;150}$ is the compressive strength tested in 150 mm cubes.

**Figure 1:**
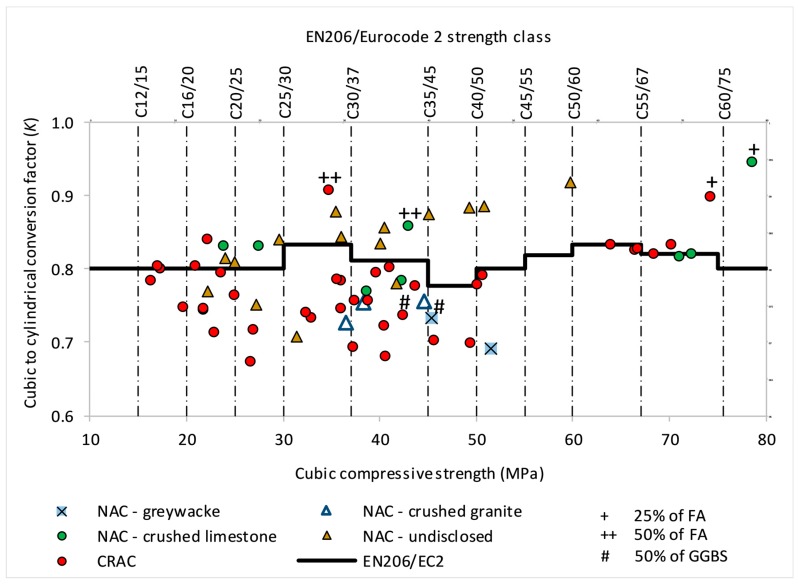
*K* versus cubic compressive strength.

The *K* versus cubic compressive strength of each composition is plotted in Figure 1, where the different types of aggregates are shown and the thick black line represents EN206/Eurocode 2’s assumption of *K*, calculated from the strength class definition of these standards. Figure 1 and the rest of the paper represent the concrete’s strength class following the notation of EN206 and Eurocode 2: CX/Y, where X refers to $f_{c,cyl\;\emptyset 150}$ and Y to $f_{c,cub\;150}$.

Table 3 shows the main statistics of *K*. The expected value of the NAC dataset (0.81) is very close to 0.80, the commonly used value in cubic to cylindrical concrete strength conversions. Full CRA incorporation reduces the average of *K* to 0.77. CRAC has lower standard deviation and coefficient of variation (CoV) than NAC, because NAC data include coarse aggregates from several origins (Figure 1), which will increase the scatter in stress-strain relationship and in ITZ quality [45], influencing micro cracking and shape effects. The CRAC database only includes coarse recycled aggregates sourced from concrete waste. This comparison may not seem fair, but for cubic to cylindrical conversion purposes, EN206 and Eurocode 2 treat all NA as the same material, and RAC has been treated worldwide as a specific type of concrete.

The influence of mix design on *K* was investigated by checking scatterplots and calculating coefficients of correlation. The coefficients of correlation between *K* and the w/b ratio and between *K* and the binder content by mass were below 0.30. The coefficients of correlation between the cubic compressive strength and *K* were equal to 0.35 (NAC) and 0.36 (CRAC).

Coefficient *K* includes factors such as the influence of plate roughness and hardness and of cylinder capping on specimen/plate friction (and subsequent multiaxial stress-states), and the effect of mould type, conservation state, and cleanliness on the specimen’s quality, geometry, and surfaces. Since these factors are not quantifiable, their influence on the value of *K* was disregarded.

## 6. Suitability of EN206/Eurocode 2 Specimen Strength Conversion

Figure 1 represents the EN206/Eurocode 2’s assumption of *K*. This was calculated after Equation (1) and assuming that concrete with cubic strength between two strength classes would be covered by the factor of the lower class (e.g., a concrete with a cubic strength of 34 MPa counted for the *K* of EN206/Eurocode 2 as a C25/30 concrete, hence its *K* is 25/30 = 0.83).

Significant scatter between the *K* of EN206/Eurocode 2 and actual data of both NAC and CRAC is observed. From Figure 1, it is observed that, when the compressive strength is below 50 MPa, the conversion factors of EN206/Eurocode 2 overestimate the strength of RAC cylinders, which may lead to unsafe designs. This means that a specific factor for RAC conversions is necessary.

The significant scatter reported for NAC is justified by a fundamental aspect of the codes: Overly complicated calculations are error-prone and cumbersome; thus, codes are intended to be as simple as possible.

**Figure 2:**
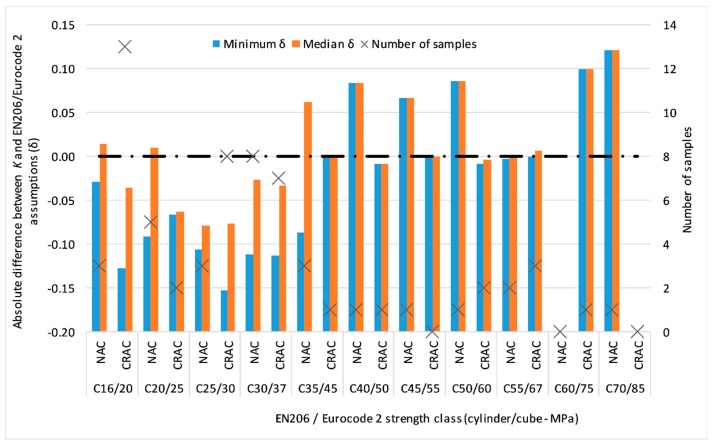
Absolute difference experimental data versus EN206/Eurocode 2 assumptions.

Since RA incorporation decreases the concrete’s compressive strength, the CRAC data of Figure 1 are biased towards lower values when compared to the data on NAC. In Figure 2, the absolute differences (δ) between the experimental values of *K* and the code assumptions are plotted and the median and minimum of δ of strength classes below the C35/45 are consistently lower for CRAC.

Three arguments are put forward for the lower *K* values of CRAC in comparison to NAC:

- The premature micro cracking of CRAC [27] limits stress-paths and the development of the fracture process zone, increasing fracture mechanics size effects;
- Ajdukiewicz & Kliszczewicz [46] found that CRA incorporation increases concrete’s Poisson’s ratio, possibly due to plastic volumetric strains [29] caused by cracking in the attached mortar of RA. Higher Poisson’s ratios lead to higher specimen/plate friction, hence higher cubic strength;
- Since RA are more heterogeneous than NA [47], statistical size effects on RAC specimens may be more meaningful. This results in a lower K because the region of the specimen under uniaxial compression on Փ 150 cylinders is higher than on 150 mm cubes (multiaxial stress-states caused by Poisson effects are more relevant near the ends of the specimens and the height of standard cylinders is twice their width).

These arguments lose relevance when the strength class increases because the relative difference between cylindrical and cubic concrete compressive strengths decreases due to higher brittleness and failure will comprise trans-aggregate fractures for RAC and NAC [7,33,48].

## 7. Definition of *K* for NAC and CRAC

**Figure 3:**
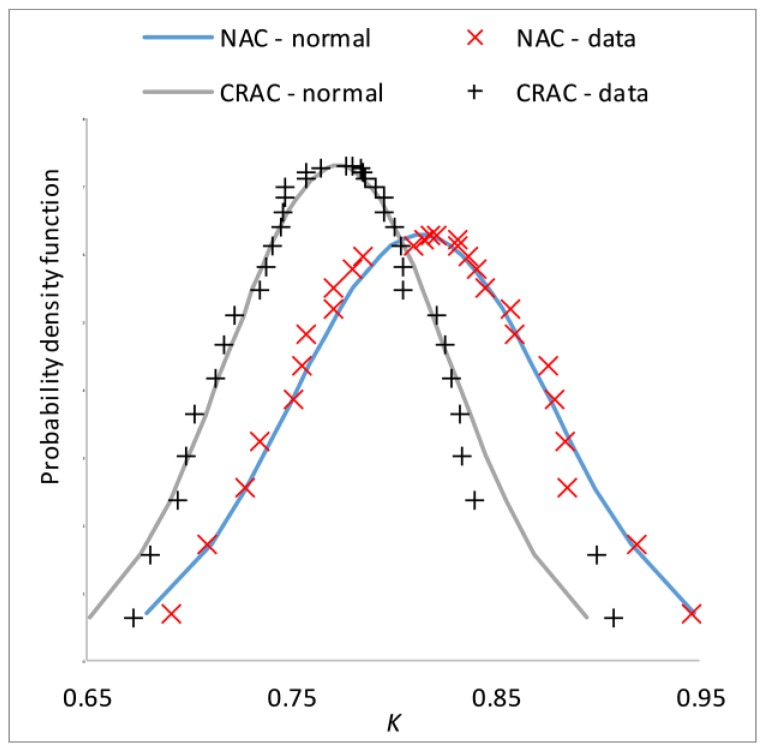
Probability goodness-of-fit.

Probability goodness-of-fit tests were performed and it was found that *K* is suitably modelled by normal distributions (Figure 3).

The statistical difference between the NAC and CRAC datasets was evaluated by the Welch *t*-test [49]. The two-tailed p-value was 0.0189, confirming that the *K*’s of NAC and of CRAC are statistically different.

Since Figure 2 showed that δ depends on the compressive strength, Table 4 evaluates the statistics of *K* for different cylinder strength classes. To ensure proper sample sizes, the strength classes were grouped two-by-two.

Higher compressive strengths result in higher average *K* factors, as is well known for NAC high-strength concrete [7]. However, a single *K* factor for all strength classes is proposed due to practicability. Moreover, *K* may be taken as independent of the compressive strength since:

- The NAC and CRAC statistics of Table 3 are within a reasonable margin of the strength-specific data of Table 4;
- When the expected values of *K* of Table 3 overestimate the strength-dependent average *K* of Table 4, the standard deviation is underestimated, offsetting this effect.

Two versions of *K* are presented: A conservative deterministic version for design and strength assessment; and a probabilistic factor for probabilistic calculations with data coming from tests on 150 mm cubes, including reliability analyses. Both versions of *K* are shown in Table 5. The deterministic conservative estimate of *K* is the lower bound of the 95% confidence interval of its expected value. If a concrete composition deviates significantly from the strength ranges or mix design parameters shown in Figure 1 and Table 2, experimental tests are recommended to check the validity of the factors proposed.

## 8. Conclusions

The relationship between the compressive strength of 150 mm cubes and Փ 150 mm × 300 mm cylinders of recycled aggregate concrete was investigated and a conversion factor proposed for full recycled coarse aggregate incorporation of aggregates sourced from concrete waste. A conversion factor for analogue compositions of natural aggregate concrete was also presented. The reasoning for different size and shape effects of recycled aggregate concrete in comparison to conventional concrete was argued for and it was statistically found the conversion of the compressive strength of recycled aggregate concrete from cubic to cylindrical specimens requires a specific conversion factor.

Both the factor for natural aggregate concrete and the factor for recycled aggregate concrete are appropriately modelled by normal distributions. The factors for conventional concrete and for recycled aggregate concrete were compared with EN206/Eurocode 2 assumptions of concrete strength classes. Below cubic compressive strengths of 50 MPa, EN206/Eurocode 2 conversions overestimate most of the cylinder strength data, especially in the case of recycled aggregate concrete and the proposed factor addresses this overestimation.

Deterministic and probabilistic versions of the factor were proposed, the first for practical conversions of tests data, and the second for reliability analyses, including code calibration. Since the actual conversion factor is dependent on concrete properties, and the concrete mix designs and materials appraised were diverse, additional tests to confirm the validity of the proposed factor are recommended. Moreover, if an unmistakeable standard compressive strength estimation is necessary, the authors recommend that the compressive strength is tested on cylinders.

## Figures and Tables

**Table 1:** Number and origin of the mixes analysed.

Research	Number of NAC Mixes	Number of CRAC Mixes	NA Type	Dmax (mm)	Number of Cubes	Number of Cylinders	Test Standard
Pedro et al. [35]	6	12	Limestone	22.4	5	2	[36]
Paul [37]	2	3	Greywacke	19	5	5	[38]
Gull [39]	3	6	Undisclosed	20	3	3	[40]
Santos et al. [41]	2	2	Limestone	20	3	2	[36]
Kheder & Al-Windaw [29]	10	10	Gravel	20	3	3	[40]
Rao & Desai [42]	3	3	Granite	20	3	3	[43]
Muhammad & Sankaranarayanan [44]	2	2	Undisclosed	20	3	3	[43]
Total	28	38	-	-	-	-	

Dmax is the maximum aggregate diameter.

**Table 2:** w/b ratios, binder contents, and cubic compressive strength of the mixes.

Relative Percentage of:	w/b Ratio			Binder Content (kg/m^3^)				Cubic Compressive Strength (MPa)		
Range	0.32–0.45	0.45–0.65	0.65–0.86	0.32–0.45	0.45–0.65	0.65–0.86	0.32–0.45	0.45–0.65	0.65–0.86	0.32–0.45
NAC	25%	61%	14%	11%	44%	38%	11%	14%	53%	32%
CRAC	24%	58%	18%	11%	48%	33%	5%	26%	47%	26%

**Table 3:** Statistical analysis of *K*.

Parameter	NAC	CRAC
Sample	28	38
Average (MPa)	0.81	0.77
Standard deviation (MPa)	0.065	0.055
CoV	8.0%	7.1%

**Table 4:** Statistics of *K* versus strength class.

Strength Class								
Statistic	C12/16 + C16/20		C20/25 + C25/30		C30/37 + C35/45		≥C45	
	NAC	CRAC	NAC	CRAC	NAC	CRAC	NAC	CRAC
Count	3	13	8	10	12	9	5	6
Average	0.81	0.76	0.77	0.74	0.82	0.78	0.88	0.84
Standard deviation	0.031	0.046	0.048	0.035	0.064	0.063	0.057	0.030
CoV	3.9%	6.1%	6.3%	4.7%	7.8%	8.1%	6.5%	3.5%

**Table 5:** Proposed deterministic and probabilistic models of *K.*

Model	Parameter	NAC	CRAC
Deterministic factor	Average	0.81	0.77
	Conservative factor	0.79	0.76
Probabilistic model (Normal)	Average	0.81	0.77
	Standard deviation	0.065	0.055
